# Conceptual Confusion: A Barrier to Multi-Professional Involvement in Advance Care Planning in Nursing Homes – An Ethnographic Study

**DOI:** 10.1177/08258597241305846

**Published:** 2024-12-18

**Authors:** Nicola Andrews, Michelle Myall

**Affiliations:** 1 School of Health Sciences, 7423 University of Southampton, Southampton, UK

**Keywords:** advance care planning, nursing homes, conceptual confusion, ethnography, multi-disciplinary team

## Abstract

**Objectives:**

How health and social care professionals need to work together to deliver advance care planning (ACP) in nursing homes is not fully understood, with a reliance on professionals external to the nursing home to support ACP in the United Kingdom. The objectives of this study were to (a) examine the factors that influence multi-professional involvement in the ACP process within nursing homes and (b) explore how multi-professional working impacts the ACP process in nursing homes.

**Methods:**

Using ethnography, data was collected through observation, interviews and document review from 36 participants including residents (*n* = 6), relatives (*n* = 4), nursing home staff (*n* = 19) and visiting professionals (*n* = 7). Data analysis combined thematic analysis, mapping of ACP trajectories for participant residents, and documentary analysis of nursing home policies.

**Results:**

There was conceptual confusion around ACP. How ACP was understood and what was prioritised for inclusion varied between residents and professionals, and between different professionals. That ACP was frequently integrated with routine care planning was not recognised in how professionals accounted for their ACP practice. Professionals prioritised biomedical concerns, despite this not reflecting resident priorities and policy suggesting a broader definition. This created difficulties in enacting ACP, with a holistic understanding of resident wishes not always captured.

**Conclusions:**

A shared understanding of ACP was not consistently evident from those tasked with its enactment. This, combined with professional construction of ACP in biomedical terms, limits multi-professional working and can prevent a person-centred process being achieved for nursing home residents.

## Introduction

In the United Kingdom (UK) care homes are increasingly providers of end-of-life care, with more than a fifth of deaths in England occurring in care homes.^1^ Nursing homes are care homes with registered nurses on site. Length of stay data is not routinely collected specifically for nursing homes but research data published in 2011 estimated the median length of stay for nursing home residents to be 1.2 years,^2^ and more recent data suggests that this duration has fallen since then for older people in care homes generally.^3^ As end-of-life care has been defined as care in the last 12 months of life,^4^ most nursing home residents are receiving end-of-life care.

Advance care planning (ACP) is intended to be a process of person-centred discussion between the person, family and care providers to identify and document goals and preferences for future care.^5–7^ It is integral to high quality end-of-life care provision^8^ and as such is considered important for nursing homes to engage with.^9,10^ ACP conversations may address issues that affect multiple dimensions of human experience, requiring expertise from a range of professional disciplines.^11^ Previous research has highlighted the importance of multi-professional involvement in ACP in nursing homes.^12^

In the UK, professionals providing care in nursing homes other than nursing staff are almost always visiting professionals,^13,14^ with the quality of end-of-life care provided contingent on the quality of a home's interrelationships with professionals from the wider health and care system.^15,16^ Nursing homes rely on external professionals to support ACP.^17,18^ However, little is known about how professionals should best work together to deliver ACP in this setting. Power imbalance between nursing home staff and visiting professionals and the negotiation of role boundaries can negatively impact multi-professional working and limit integration of nursing homes in system-wide approaches to ACP.^19^

Previous research on multi-professional involvement in ACP within nursing homes is limited. A Canadian study exploring interprofessional staff perceptions of ACP in long-term care concluded that interdisciplinary collaboration is required, and identified structures required to support this.^20^ This paper reports findings from a doctoral study that specifically examined the impact of multi-professional involvement in ACP within the nursing home setting.^21^ To our knowledge, this is the first study to explore multi-professional working through the vehicle of ACP in nursing home settings. The study addressed the research questions: (a) What factors influence multi-professional involvement in the ACP process within nursing homes? (b) How does multi-professional working impact the ACP process in nursing homes? The definition of ACP adopted for the study was the accepted definition of ACP in the UK at the outset of the research^22^ extended to incorporate processes by which an individual's expressed wishes are implemented, based on research suggesting multi-professional involvement is significant in both ascertaining and implementing nursing home residents’ wishes.^17^

## Methods

### Study Design

An ethnography was conducted to understand meanings motivating the actions of residents, relatives and professionals in ACP in two UK nursing homes.^23^ Using non-participant observation, interviews and document review, social interactions underpinning multi-professional working were explored. The study was approved by the Social Care Research Ethics Committee (15/IEC08/0004).

### Settings and Participants

Two nursing homes with variation in key characteristics (Table 1) were purposively sampled via specialist palliative care education facilitators. NA visited the homes and shared information about the study prior to fieldwork commencing.

**Table 1.** Nursing Home Characteristics.

	Nursing Home 1	Nursing Home 2
Number of beds	More than 50 beds; mixture of with and without nursing care.	More than 50 beds; all residents receiving nursing care.
Location	Urban	Rural
Ownership	Large private care home organisation with 20+ homes.	Small private care home organisation with <9 homes.
Length of stay	Short stay admissions (respite/convalescence) common, with at least 13 during fieldwork.	Two residents had respite (short stay) admissions during fieldwork. Two had lived in the home for 12 years.
Staffing	Minimum of two nurses during the day and evening, and one nurse overnight. The organisations’ other homes provided support when needed, with agency staff used infrequently.	Minimum of two nurses both day and night. Infrequent use of agency staff.
Multi-professional services	All residents registered with one of three GP practices. One main practice (where 75%–85% of residents registered at any one time). NHS provision, not privately contracted.	One GP practice, contracted to provide medical services above usual NHS care provision including a weekly GP round, provided care to most residents. 10%–15% at any one time registered with the GP practice located closest to the home.

A purposive sample including nursing home staff, residents, relatives and visiting professionals was recruited. Inclusion and exclusion criteria are provided in Table 2. Prior to recruitment, all potential participants received written information, either by post or via nursing home staff, providing full details about the study. Residents approached had engaged with ACP to different extents, had diverse visiting professional involvement and varied prognoses. Nursing home managers and nurses assisted with identification of residents and staff to approach. Visiting professionals involved in the care of and relatives of residents recruited were invited to participate.

**Table 2.** Inclusion and Exclusion Criteria for Participants.

Participant type	Inclusion criteria	Exclusion criteria
Residents	Living in a participating nursing home. Previously completed some ACP.	Lacking mental capacity either to be involved in ACP or to consent to take part in the study.
Relatives	Nominated by a participating resident.	
Nursing home staff	Involved in ACP activities in a participating nursing home, providing care to a participating resident or identified as a key stakeholder.	Not employed by a participating nursing home.
Visiting health and social care professionals	Providing care to residents living in a participating nursing home. Involved in key ACP activities in a participating nursing home or involved in the care of a participating resident.	

Six residents, four relatives, nineteen nursing home staff and seven visiting professionals provided written, informed consent and participated in the study (Table 3). Residents included men (*n* = 3) and women (*n* = 3), aged between 79 and 93. Relatives were all adult children of residents.

**Table 3.** Numbers Approached to Participate and Recruited.

Participant group	Approached	Recruited
Residents	22	6
Relatives	6	4
Nursing home managers	4	4
Nursing home nurses	21	6
Nursing home care staff	15	8
Nursing home activities staff	2	1
GPs	8	3
Specialist nurses	4	3
Social care professionals	1	1

### Data Collection

More than 200 hours of fieldwork were undertaken by NA, who spent between 6 and 7 months in each setting. This involved more than 50 separate visits to each nursing home, including day, night and all days of the week. NA worked concurrently as a specialist palliative care nurse and kept a reflexive diary to support identification of tacit knowledge informing interpretation. Participant behaviour may also have been influenced by this as they were informed about NA's clinical role in the participant information sheet.

Observations focused on events involving multi-professional working, recorded in field notes. Daily routines of nursing home staff and discussions relating to care provision between nursing home staff or nursing home staff and visiting professionals were observed. Observations also included interactions between nursing home staff and/or visiting professionals with participating residents or relatives. Observations were unstructured, without use of an observation framework. However, decisions for prioritising the focus for observations were, at least initially, informed by NA's nursing experience.

NA completed 17 audio-recorded interviews of between 25 and 55 minutes duration, 16 individual interviews and one with a resident-relative dyad. Informal interviews, characterised by researcher-led questions asked while observing, were used when formal interviews were not possible, most often due to lack of time. These differed from naturally occurring talk where questions were led by the situation being observed. All interviews explored views and experiences of ACP and multi-professional involvement in ACP, and barriers and facilitators of multi-professional involvement.

With informed consent of the manager, nursing home documents such as end-of-life care policies were reviewed, as were resident notes with permission of the resident. Documents were re-read at intervals to identify any updates.

### Data Analysis

**Figure 1.**
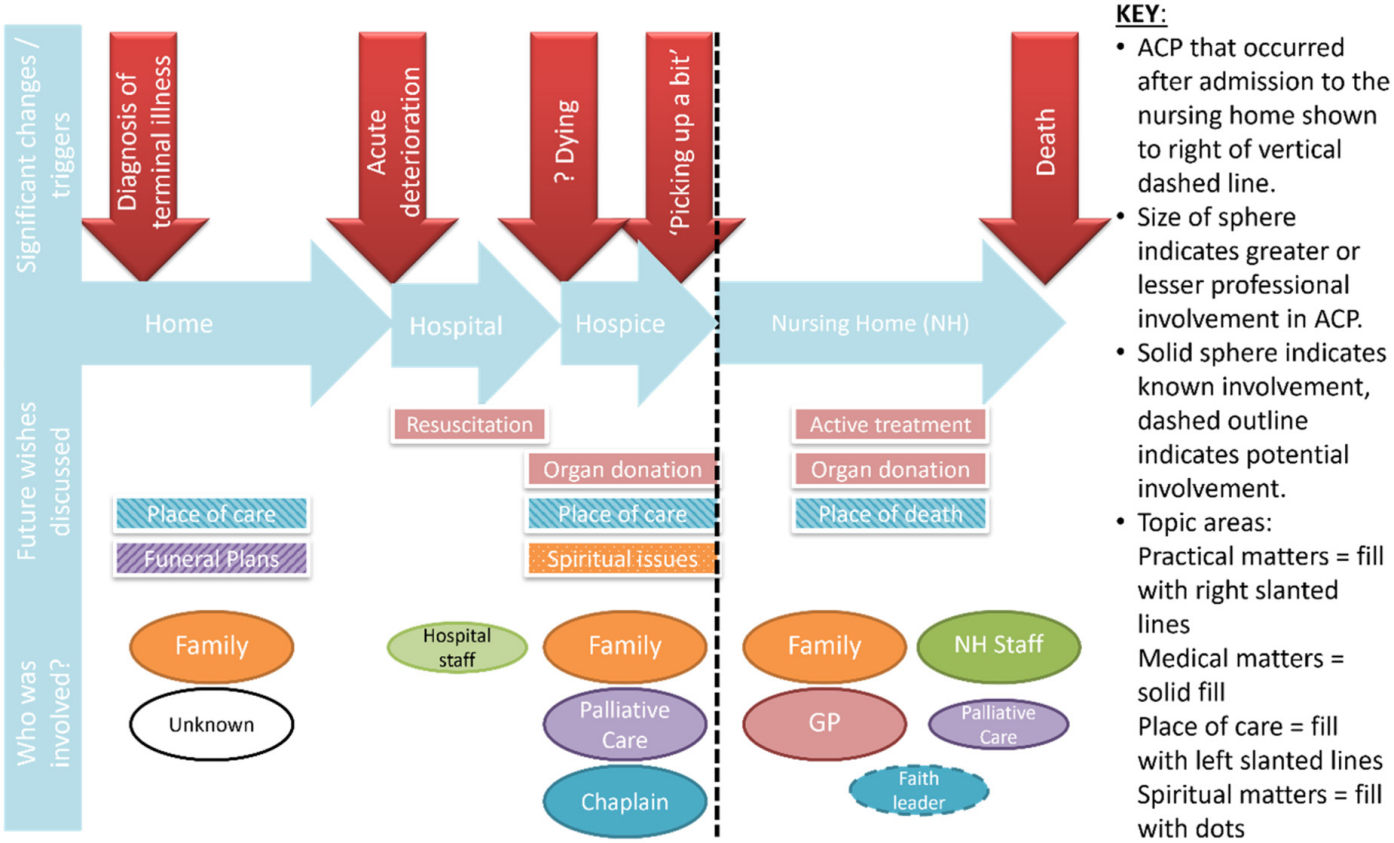
Example map of a resident's ACP trajectory.

Analysis was inductive and iterative and involved three strands. Firstly, thematic analysis using Braun and Clarke's approach.^24^ Secondly, data relating to each resident's ACP trajectory was mapped (an example is provided in Figure 1). Thirdly, documentary analysis of nursing home policies. Initial coding of all observation, interview and document review data, mapping of trajectories and documentary analysis was completed by NA. These analyses were then integrated and themes evolved through discussion with MM. This combined individual resident and organisational perspectives to produce a rich ethnographic picture of multi-professional working in relation to ACP. Data was managed using NVivo (version 11) and Microsoft Excel.

## Results

This article presents three themes identified through the analyses relating to the conceptualisation of ACP and the impact of this on enacting ACP in practice. Pseudonyms are used throughout.

### Marginalisation of the Psychosocial-Spiritual in ACP

Residents’ framing of future planning differed from that of professionals. Residents prioritised concerns of a practical or personal nature and often had not considered decisions relating to treatment. Professionals primarily conceptualised ACP in biomedical terms, which led to operationalisation of ACP in terms of medical decisions relating to end-of-life care. Treatment escalation and resuscitation, in particular, dominated professional talk about ACP.

Hilda [resident] said she had been quite detailed in some of her funeral plans, such as she had chosen the picture to go on the order of service. (Field notes – visit 046)

‘If there isn’t documentation for end-of-life decision-making such as do not resuscitate obviously you need to engage with that’. (Sandra, Specialist nurse)

Information selected for recording in residents’ notes evidenced the dominant biomedical approach to professional-led ACP. For participating residents, the sections related to medical matters in ACP documents were completed more often, only two plans included brief reference to psychosocial or spiritual issues. Yet, all six talked about psychosocial or spiritual matters that influenced their future preferences, such as bereavement and existential concerns.

Lily [resident] talked about not wanting to go back to hospital but that she is aware that they can’t do things in the nursing home that they can in hospital. She said she wasn’t sure if she wants to be “saved” but is clear that she doesn’t want to find herself “ga-ga” as she describes it. (Field notes – Visit 085)

In one nursing home, activity coordinators gathered life history information which could have informed understanding of residents’ values and wishes beyond the biomedical sphere. However, their role did not extend to documenting in the home's ACP record, which was completed by nursing staff only. This suggests psychosocial contributions to ACP were considered of lower priority than information recorded about specific treatment decisions. Yet ACP policy in both homes indicated that ACP should incorporate psychosocial and spiritual wishes.

### Timing of ACP Conversations

Residents’ planning, focused on personal matters relevant to them, often commenced before their move into residential care, whereas professional construction of ACP in medical terms, whereby conversations were treatment focused, often constrained when they were initiated. The idea of a ‘right time’ to have discussions about future treatment and care was a widely held perception among residents, relatives and professionals alike.

‘With our policy it says that as soon as possible we have to actually discuss that. But the timing should actually be right I think with that aspect’. (May, Manager)

Residents rarely instigated discussions about future treatment, not wanting to think about this until their condition deteriorated.

‘[…] if I was really feeling ill, I’d probably start making plans for, you know, what's going to happen to me, but it hasn’t got to that stage. […] I haven’t thought about it even’. (Jim, resident)

Making decisions relating to care and treatment in advance was also difficult for residents due to uncertainty about health concerns they might face in the future.

Christine [nursing home nurse] said discussions are easier when there is something concrete to think about. […] it is difficult to think about what care you may or may not want when you don’t know what you may or may not need. (Field notes – Visit 058)

One resident described changing her mind, after receiving treatment she had not anticipated and regretted undergoing:

‘They gave me blood transfusions. I wish they’d never done it. But I wasn’t in a fit state to refuse anything, but I certainly would never want it again’. (Mabel, resident)

It was therefore the role of professionals to open these conversations, but it was evident that they felt more comfortable doing this when a resident's condition deteriorated. This provided them with an identified reason for discussing ACP, without which the perception was that it risked upsetting residents and/or their relatives. However, residents had unpredictable trajectories to the end-of-life, observed both becoming unwell and dying quickly and recovering when death was expected with near certainty. Aligning discussions about future medical needs was therefore challenging.

### Lack of Shared Understanding

Residents did not always recognise when they had been involved in ACP. In one home, two residents remembered completing an ACP form, but not all future planning involved separate documentation or standalone processes. For example, five of the participating residents discussed some aspects of ACP as part of general care planning. One relative could not identify future planning that had taken place, despite ACP completed at a care plan review documented in her father's notes:

‘I had quite a few meetings […] just about his general care and settling in and so on. But I don’t think we’ve actually talked particularly about any planning as such’. (Jackie, relative)

Likewise, professionals understood specifically arranged discussions to complete ACP documents as ACP but did not always recognise future care planning when it was integrated within wider care planning. This was despite one home's ACP policy explicitly linking ACP and care planning. A visiting professional revised her initial view that she was not involved with ACP after talking through how she managed symptoms, identifying how frequently this would include discussion of future preferences:

‘I’d say to them you know it's not safe to do this here and the safer place would be to do it in hospital […] and then you would go on and have that discussion with them. […] So then yes, I would I suppose get involved’. (Lynne, Specialist nurse)

This professional's initial interpretation of her involvement with residents’ ACP was shared by one of the nursing home nurses and a GP.

‘No. She will deal with [their illness] and their medication and liaise with the hospital consultants but she's not involved in advance care planning’. (Dr Slater, GP)

Blurring of ACP with care planning could therefore lead to future wishes included in information routinely shared with other professionals after her visits not being documented as ACP.

## Discussion

Through use of ethnography, this study has provided rich insights into the social processes that influence multi-professional involvement in ACP in two nursing homes. The findings show conceptual confusion surrounding ACP, with a lack of consistency in how it was defined, perceived or enacted by those involved, offering new insight into the complexity of ACP. The process of ACP functioned as an organisational routine and using this as a theoretical lens on how actors jointly accomplish the interdependent task of ACP, highlights the differences between the ostensive, performative and proxy aspects of the routines of the different organisations and actors involved.^25,26^ The ostensive aspect is characterised as the way participants account for specific performances of an ACP routine, the performative aspect as the actual performance of the routine by participants, and artefacts, such as ACP policy, serve as a proxy for the ostensive or performative routines.^26^

The findings show that the ostensive aspect of professionals’ ACP routines constructed it as something distinct from other care planning, such as separate ACP discussions and specific ACP documentation. Yet, professionals’ performative routines often integrated ACP with day-to-day care planning*.* Although early UK guidance outlined ACP as part of the wider care planning process^22^ and research has found ACP in practice is integrated with other end-of-life planning and care discussions,^27^ the inter-relationship between ACP and care planning is not explicit in ACP definitions. This means that professionals might interpret some expressed wishes as ACP, some as routine care planning, and these might not be integrated into a holistic representation of a resident's future wishes.

In both nursing homes, the ostensive and performative routines also differed from the proxy routines in terms of what was identified as ACP. Despite ACP policy in both homes suggesting a broader definition of ACP, what was documented and discussed as ACP focused on biomedical concerns. The need to contextualise ACP within a person's individual life, exploring their values and goals rather than specific treatment decisions has been widely reported.^28–30^ Yet, professionals’ construction of ACP did not situate the resident within their social world or seek to understand what was important to them beyond a primary focus on biomedicine, findings also reported elsewhere.^30^

Although religious ministers were not invited to record in residents’ notes in either of the homes, this reflects that clergy rarely share information due to the strict confidentiality of their encounters.^31^ Yet research suggests that many religious ministers facilitate fulfilment of preferred spiritual care,^32^ although spiritual matters are largely absent from ACP literature.^33^ However, activity coordinators similarly did not record their knowledge of residents’ wishes and values in the ACP documents. What was recorded as ACP may therefore not have reflected residents’ priorities. This supports previous research findings suggesting a need to deemphasise the biomedical approach and consider the aspects prioritised by residents.^29^

Ambiguity about the meaning of ACP and what it comprises exists,^34^ with research findings indicating that it is not universally understood or interpreted.^27,30,35–37^ Yet that ACP can be understood in different ways is not evident in ACP literature, with Froggatt et al^17^ purporting the term is often used without definition or explanation. This study has shown that this conceptual confusion can create difficulties enacting ACP. The different meanings given to ACP by both professionals and residents means expressed wishes or preferences might not be shared as ACP across all teams and organisations involved. This could lead to them not being readily accessible to or known by professionals when needed, thereby impacting ability to honour residents’ wishes.

The findings also suggest enacting ACP is constrained by professionals’ biomedical framing of ACP affecting initiation of discussions, with the commonly cited barrier to ACP of finding the ‘right time’ primarily associated with challenges in prognostication.^37–39^ The biomedical focus created difficulties for residents when there was uncertainty about ill-health they might face in the future. Previous research suggests older people are less concerned about planning for end-of-life situations outside their imagination^40^ and consider decisions can only be made when it is clear what is being faced.^41^ However, this may happen too late, demonstrated by the unpredictability inherent in residents’ end-of-life trajectories. Indeed, the focus of residents in this study on practical and personal planning reflects previous findings suggesting older people plan for after death rather than future health needs and concentrate on living rather than dying.^41–43^ Combes et al^44^ suggest models of ACP that focus on living well now are needed for frail, older people.

### Implications for Practice, Education, Policy and Research

The study findings suggest a need for greater awareness of the overlap between ACP and care planning. Making this more explicit in definitions and policy, alongside including ACP in all education relating to care and support planning in health and social care, rather than specifically as a component of end-of-life care education, could assist in achieving this. The study has also highlighted the dominant biomedical model of ACP and how this both makes opening conversations more difficult as well as not aligning well with resident priorities. A focus on exploring an individual's values to inform future decision-making, as well as wishes outside the biomedical domain, need to be embedded into ACP practice. Further research is also required to inform development of models of integrated, multi-professional involvement that support ACP defined more broadly than in biomedical terms.

### Strengths and Limitations

A strength of the study was the use of ethnography, which provided unique insight into both the nursing home culture and healthcare culture that shape ACP in this setting. It also provided depth and breadth of insight gained through use of multiple methods to collect data from multiple stakeholder groups. A further strength was that it was not constrained by ACP definitions but investigated future planning and end-of-life care decision-making more broadly, which allowed exploration of how ACP was understood.

However, participant information explicitly linked end-of-life care and ACP, and also identified the researcher (NA) as a palliative care nurse which may have influenced participants’ behaviour and limited the depth of understanding gained from participants about the meaning they might attach to ACP. Another limitation was recruitment only of residents who had mental capacity to both complete ACP and consent to the study. No participating residents had a diagnosis of dementia, yet a substantial majority of care home residents in the UK have some cognitive impairment.^45^

## Conclusion

These findings show that the conceptualisation of ACP is not always shared by everyone with a lack of collective understanding as to what constitutes ACP both between professionals and between residents and professionals. This conceptual confusion alongside the professional construction of ACP in biomedical terms created challenges in both ascertaining and implementing ACP. These findings contribute to understanding how ACP could be enhanced so an integrated multi-professional, person-centred process can be achieved for nursing home residents.
